# Germline landscape of BRCAs by 7-site collaborations as a BRCA consortium in Turkey

**DOI:** 10.1016/j.breast.2022.06.005

**Published:** 2022-06-21

**Authors:** Atil Bisgin, Sebnem Ozemri Sag, Muhammet E. Dogan, Mahmut S. Yildirim, Aydeniz Aydin Gumus, Nejmiye Akkus, Ozgur Balasar, Ceren D. Durmaz, Recep Ersoz, Sule Altiner, Adem Alemdar, Lamia Aliyeva, Ibrahim Boga, Fethi S. Cam, Berkcan Dogan, Onur Esbah, Abdullah Hanta, Cem Mujde, Cemre Ornek, Sinem Ozer, Cagla Rencuzogullari, Ozge Sonmezler, Sevcan Tug Bozdogan, Munis Dundar, Sehime G. Temel

**Affiliations:** a Cukurova University AGENTEM (Adana Genetic Diseases Diagnosis and Treatment Center) and Medical Genetics Department of Medical Faculty, Adana, Turkey; b InfoGenom, Cukurova Technopolis, Adana, Turkey; c Bursa Uludag University, Faculty of Medicine, Department of Medical Genetics, Bursa, Turkey; d Erciyes University, Faculty of Medicine, Department of Medical Genetics, Kayseri, Turkey; e Necmettin Erbakan University, Meram Faculty of Medicine, Department of Medical Genetics, Konya, Turkey; f Manisa Celal Bayar University, Faculty of Medicine, Department of Medical Genetics, Manisa, Turkey; g Health Sciences University, Kocaeli Derince Training and Research Hospital, Department of Medical Genetics, Kocaeli, Turkey; h Health Sciences University, Konya City Hospital, Department of Medical Genetics, Konya, Turkey; i Health Sciences University, Gazi Yasargil Training and Research Hospital, Department of Medical Genetics, Diyarbakir, Turkey; j Hacettepe University, Faculty of Medicine, Department of Medical Genetics, Ankara, Turkey; k Duzce University, Faculty of Medicine, Department of Medical Genetics, Duzce, Turkey; l Health Sciences University, Kanuni Training and Research Hospital, Department of Medical Genetics, Trabzon, Turkey; m Ankara University, Faculty of Medicine, Department of Medical Genetics, Ankara, Turkey; n Bursa Uludag University, Institute of Health Sciences, Department of Translational Medicine, Bursa, Turkey; o Duzce University, Faculty of Medicine, Department of Medical Oncology, Duzce, Turkey

**Keywords:** *BRCA* profiling, *BRCA* landscape, Population study, Genomic screening

## Abstract

*BRCA1/2* mutations play a significant role in cancer pathogenesis and predisposition particularly in breast, ovarian and prostate cancers. Thus, germline analysis of *BRCA1* and *BRCA2* is essential for clinical management strategies aiming at the identification of recurrent and novel mutations that could be used as a first screening approach. We analyzed germline variants of *BRCA1/2* genes for 2168 individuals who had cancer diagnosis or high risk assessment due to BRCAs related cancers, referred to 10 health care centers distributed across 7 regions covering the Turkish landscape. Overall, 68 and 157 distinct mutations were identified in *BRCA1* and *BRCA2*, respectively. Twenty-two novel variants were reported from both genes while *BRCA2* showed higher mutational heterogeneity. We herein report the collective data as BRCA Turkish consortium that confirm the molecular heterogeneity in BRCAs among Turkish population, and also as the first study presenting the both geographical, demographical and gene based landscape of all recurrent and novel mutations which some might be a founder effect in comparison to global databases. This wider perspective leads to the most accurate variant interpretations which pave the way for the more precise and efficient management affecting the clinical and molecular aspects.

## Highlights

• A similar percentage of clinically unaffected individuals were also identified with potentially pathogenic variants.
• Based on the geographic examinations, Eastern Anatolian and the Mediterranean region had the lowest positivity rates.
• The Southeastern Anatolian region showed the lowest density of both patients and clinically relevant genetic changes.

## 1 Introduction

*BRCA1* and *BRCA2* are well known tumor suppressor genes that play significant roles in the control of homology-directed DNA damage response (DDR) for double chain break repairs. Detection of *BRCA1*/2 germline mutations is necessary for diagnosing and prophylactically preventing several types of cancers, especially those of the breast, ovary and prostate. Additionally, defining the variants that are common among particular ethnic populations may provide for better targeted clinical evaluations based on population specific genotype-phenotype relations.

**Fig. 1:**
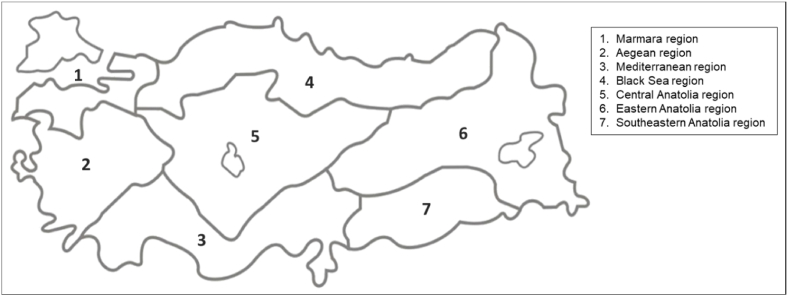
Geographic regions of Turkey (Created by using Adobe Illustrator 21.0.0) [43].

Turkey's population is comprised of many ethnicities with extensive variation with regard to geography and social strata. Due to the recent changes in Turkey's demographic structure, the genetic heterogeneity of its population has continued to increase. Therefore, evaluation of the frequencies of the various *BRCA1* and *BRCA2* alleles cannot be done based on ethnic origins. Thus, our aim was to investigate different geographic locations throughout Turkey to determine the most common cancer-related genetic variations in the *BRCA1*/2 genes based on regions. This affords us an opportunity to establish an effective genomic landscape that compares the similarities and differences among the country's major geographic areas (Fig. 1). For this study, we collected data from seven major geographic regions that included the largest cohort ever examined of the Turkish population through the participation of eight genetic diagnostic centers.

The BRCA gene mutations and their relative population frequencies have been extensively researched among European North and South American populations, (including the USA, Canada and Brazil), as well as Asian populations (including China, India and Japan) [[1], [2], [3], [4]]. The resulting information -especially the data from European, Caucasian, Ashkenazi and North American populations- is used globally as the standard references for clinical diagnoses, treatment and prevention of BRCA‐related cancers. Recent studies suggest that BRCA mutations can be ethno‐specific, raising the question of whether limited population-based information should be used as a universal standard or if a population‐based BRCA mutation information system needs to be developed. Many of the studies have emphasized the importance of revealing the population-specific BRCA variant spectrum and frequency, and show its necessity for facilitating the development of a robust risk prediction calculator for the various populations [5,6].

The lack of comprehensive, in depth research regarding the Turkish BRCAs mutations and their frequencies was obvious foundation/basis of this study and based on the ever-growing Turkish population because of both shifting demographics within the country and immigration. Previous studies of *BRCA1*/*BRCA2* mutational frequencies performed in Turkey were prior to the wide-spread adoption of next generation sequencing (NGS)-based studies that provide a comprehensive mutational spectrum analyses that provides and thus were limited to a relatively few known variants [[7], [8], [9], [10], [11], [12]].

In this study, we assembled the *BRCA1* and *BRCA2* short-read NGS, Sanger sequencing and MLPA retrospective results of 2168 individuals who applied or were referred to our consortium centers across Turkey from 2014 to 2020. Patients who were assessed to be in the high risk group for disease susceptibility or had a diagnosis of relevant types of cancers such as breast and endometrial cancer were enrolled in the study. In addition, genetic screening of clinically unaffected individuals was included to the cumulative data.

Here we present our multi-center data describing the Turkish *BRCA1*/2 germline mutation landscape which can be used to aid in region-specific diagnostics to provide for more accurate patient management through preventive medicine and genetic counseling, as well as adding to the global literature on the subject.

## 2 Material and methods

### 2.1 Demographic information

The Turkish population is comprised of a multiple and mixed ethnic groups due to its location as a bridge between Asian and European people and the impacts from major geopolitical events. In this study, NCCN Clinical Practice Guidelines in Oncology were followed for the cases, and ACMG criteria were implemented for healthy individuals at increased risk of a hereditary cancer syndrome [13,14]. Clinically unaffected individuals who have a first- or second- degree blood relative meeting any of the NCCN and ACMG criteria were included to the study group, while individuals from the same family were excluded from the cumulative data to support the rationale of our study approach. The mutational results of *BRCA1*/2 screening from 2168 individuals who applied or were referred to centers in our consortium were investigated retrospectively. Region-based clinical distribution of individuals were given in Supplementary Table 2. Informed consent was obtained from all participants according to the terms of the Helsinki declaration. Collected data were evaluated in terms of the patients' diagnoses and their risk group if from the clinically unaffected individuals.

### 2.2 Sampling and genetic material isolation

Peripheral blood samples were obtained from clinically unaffected individuals and patients with cancer diagnosis. Genomic DNA (gDNA) was isolated from all peripheral blood samples using QIAcube and QIAsymphony platforms (Qiagen, Germany). The quality and the quantity of the DNAs were assessed fluorometrically. Samples that did not meet the quality criteria were excluded.

### 2.3 Next generation sequencing

All exons and exon-intron junctions of the *BRCA1*/2 genes were subjected to next generation sequencing via one of three different NGS devices: GeneReader NGS System (Qiagen Germany), MiSeq and NextSeq 550 (Illumina, USA) platforms.

### 2.4 Sanger sequencing

Sanger sequencing was performed using a genetic analyzer (Thermo Fisher Scientific, Waltham, Massachusetts USA) according to the manufacturer's instructions.

### 2.5 MLPA (Multiplex ligation-dependent probe amplification)

The MLPA method was performed using a genetic analyzer (Thermo Fisher Scientific, Waltham, Massachusetts, USA). The copy number was calculated according to the MLPA kit instructions. A relative peak-height of less than 0.8 was defined as a “Homozygous deletion”, between 0.8 and 1.25 as “normal”, and between 1.75 and 2.15 as a “Homozygous duplication”.

### 2.6 Bioinformatics and variant classification

Bioinformatics analyses were performed comparatively with both population and clinical databases (including gnomAd, exAc, 1000 genomes, Ingenuity Knowledge Base, ClinVar, HGMD, OMIM, Genetic Home Reference, dbSNP and VarSome). Variants were classified according to ACMG criteria by taking BRCAexchange database into consideration. Benign and likely benign genetic changes were filtered out. Hg19 [Genome Reference Consortium Human Build 37 (GRCh37)] as the reference sequence, RefSeq transcripts NM_007294.4 for *BRCA1* and NM_000059.4 for *BRCA2* gene were used for variant nomination accordingly with HGVS (Human Genome Variation Society) nomenclature.

## 3 Results

**Fig. 2:**
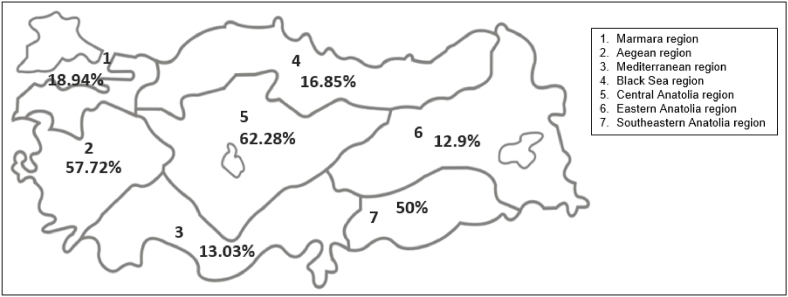
Regional distribution of positivity rates in *BRCA1* and *BRCA2* genes (Created by using Adobe Illustrator 21.0.0) [43].

From the 2168 patients enrolled in the study, 1655 had a diagnosis of cancer prior to genetic testing. Among them, 342 (20.66%) of these cancer patients had *BRCA1*/2 variants associated with disease. A similar percentage, 22.61% (116/513) of clinically unaffected individuals were also identified with potentially pathogenic variants. Based on the geographic examinations the highest positivity rate was seen in the Aegean and Central Anatolian regions, while the Eastern Anatolian and the Mediterranean region had the lowest positivity rates. The regional distributions of the positivity rates of cancer patients and clinically unaffected individuals are shown in Fig. 2.

Among the 2168 patients, we detected (n = 471) clinically significant variants (Pathogenic, likely pathogenic variants) in 317 individuals for an overall a positivity rate of 14.6% while 137 patients carry only variants of uncertain significance (VUS). We also assessed MLPA for the detection of microduplications/microdeletions in all *BRCA1/2* negative patients. Controversially to the literature, our MLPA studies revealed no deletion or duplication in any cases. Thirteen patients had more than one genetic alterations in either one of both *BRCA* genes (Table 1). The mutation detection rate of the study was 21.72% with 471 reported variants. Among the 471 detected variants, there were 225 different genetic alterations with 39.7% (n = 187) found in the *BRCA1* gene, and 284 (60.3%) identified in *BRCA2*.

**Table 1:** Variant distribution of patients carry more than one mutation in either *BRCA1* or *BRCA2* genes.

**More than one variation in *BRCA1***		
	**Genetic alteration 1**	**Genetic alteration 2**
P1	c.1444_1447delATTA p.I482*	c.5123C > A p.A1708E
P2	c.4065_4068delTCAA p.N1355Kfs*10	c.2599C > G p.Q867E
**More than one variation in *BRCA2***		
	**Genetic alteration 1**	**Genetic alteration 2**
P3	c.1414C > T p.Q472*	c.8359C > T p.R2787C
P4	c.1909 + 22delT	c.1343G > A p.R448H
P5	c.4587_4588insA p.V1532Sfs*2	c.1146A > T p.K382N
P6	c.8020_8021dupAA p.I2675fs*2	c.6080G > A p.R2027K
P7	c.1235C > G p.P412R	c.4081C > G p.Q1361E
P8	c.1235C > G p.P412R	c.4081C > G p.Q1361E
**Both *BRCA1* and *BRCA2***		
	***BRCA1* variant**	***BRCA2* variant**
P9	c.1444_1447delATTA p.I482*	c.1411G > A p.E471K
P10	c.4956G > A p.M1652I	c.3836A > G p.N1279S
P11	c.981_982delAT p.C328*	c.658_659delGT p.V220fs*4
P12	c.4956G > C p.M1652I	c.6614T > G p.V2205G
P13	c.3247A > G p.M1083V	c.9097dupA p.3033fs*11

Variant classification assessments for all detected variants were made according to the ACMG and Association for Molecular Pathology (AMP) criteria, and the pathogenicity classes that were included in our study were: pathogenic, likely pathogenic, and VUS. We excluded the benign and likely benign variants. The distribution of variant pathogenicity profiles for *BRCA1* and *BRCA2* are shown in Table 2. Overall, most of the VUS (n = 116) were detected in cancer patients while only 28 were observed in clinically unaffected individuals resulting in similar ratio (5.06% for cancer patients, 5.03% for unaffected individuals).

**Table 2:** Pathogenicity distribution of detected different variants in *BRCA1* and *BRCA2* genes.

	Pathogenic	Likely pathogenic	VUS
*BRCA1*	39	6	23
*BRCA2*	54	10	93

Patients with detected variants were further investigated in terms of regional geographic distribution. The Mediterranean, Marmara and Central Anatolian regions differed from other regions with a higher number of patients and identified variants. Variants and patients' regional distributions are shown in Table 3. The Southeastern Anatolian region showed the lowest density of both patients and clinically relevant genetic changes.

**Table 3:** Regional geographic distribution of *BRCA1*/2 variant detected based on clinical presentation.

	Cancer Diagnosis	Screening of clinically unaffected individuals
Mediterranean Region	119	46
Aegean Region	29	–
Black Sea Region	15	–
Central Anatolia Region	79	63
Marmara Region	77	9
Eastern Anatolia Region	8	–
Southeastern Anatolia Region	13	–

Frequency and pathogenicity of recurrent variants that were detected in more than one patient are shown in Table 4.

**Table 4:** Pathogenicity distribution of detected pathogenic and likely pathogenic variants in the *BRCA1* and *BRCA2* genes.

Gene	n	Genetic alteration		Internal Freq.	Pathogenicity
*BRCA1*	20	c.1444_1447delATTA	p.I482*	0.0092	P
*BRCA1*	17	c.5266dupC	p.Q1756Pfs*74	0.0078	P
*BRCA1*	16	c.2800C > T	p.Q934*	0.0074	P
*BRCA1*	13	c.4327C > T	p.R1443*	0.0060	P
*BRCA1*	7	c.5123C > A	p.A1708E	0.0032	P
*BRCA1*	7	c.181T > G	p.C61G	0.0032	P
*BRCA1*	6	c.981_982delAT	p.C328*	0.0028	P
*BRCA1*	5	c.4035delA	p.E1346fs*20	0.0023	P
*BRCA1*	4	c.2611_2612delCC	p.P871Vfs*31	0.0018	P
*BRCA1*	4	c.3211G > T	p.E1071*	0.0018	P
*BRCA1*	4	c.3607C > T	p.R1203*	0.0018	P
*BRCA1*	4	c.4391_4393delinsTT	p.P1464Lfs*2	0.0018	P
*BRCA2*	9	c.2765dupT	p.K923Qfs*13	0.0042	P
*BRCA2*	9	c.9097dupA	p.T3033Nfs*11	0.0042	P
*BRCA2*	8	c.7689delC	p.H2563Qfs*85	0.0037	P
*BRCA2*	6	c.3751dupA	p.T1251Nfs*14	0.0028	P
*BRCA2*	6	c.4169delT	p.L1390Wfs*20	0.0028	P
*BRCA2*	6	c.67+1G > A	–	0.0028	P
*BRCA2*	5	c.7976G > A	p.R2659K	0.0023	P
*BRCA2*	5	c.1773_1776delTTAT	p.I591Mfs*22	0.0018	P
*BRCA2*	4	c.1519delA	p.R507Efs*2	0.0018	LP
*BRCA2*	4	c.5969delA	p.D1990Vfs*14	0.0018	P
*BRCA2*	4	c.7007G > A	p.R2336H	0.0018	P
*BRCA2*	4	c.8478C > A	p.Y2826*	0.0018	P

The frequencies of the detected variants within this study show differences from the population databases that are in global use. The genetic changes that have higher frequencies than the global population databases are shown in Supplementary Table S1.

**Fig. 3:**
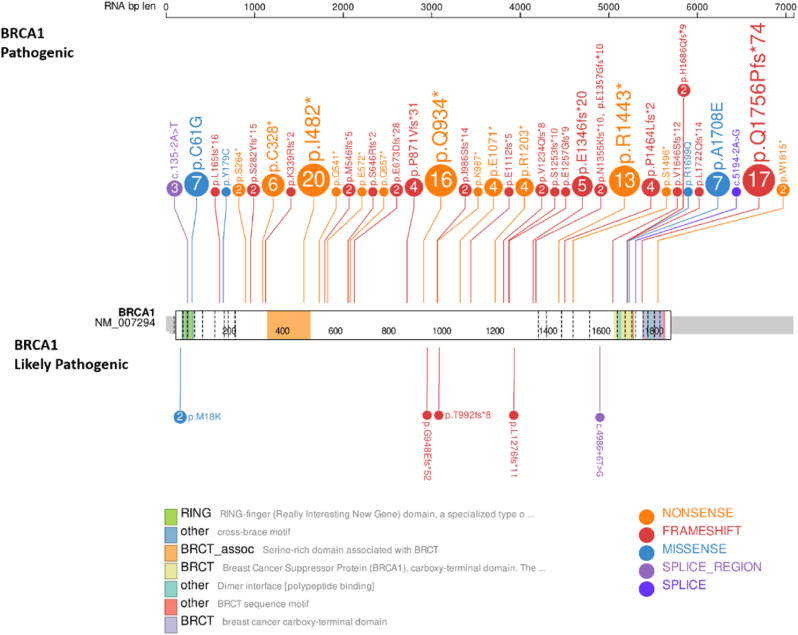
The detected pathogenic and likely pathogenic mutations in *BRCA1* gene (Created by using ProteinPaint, 2021) [44].

**Fig. 4:**
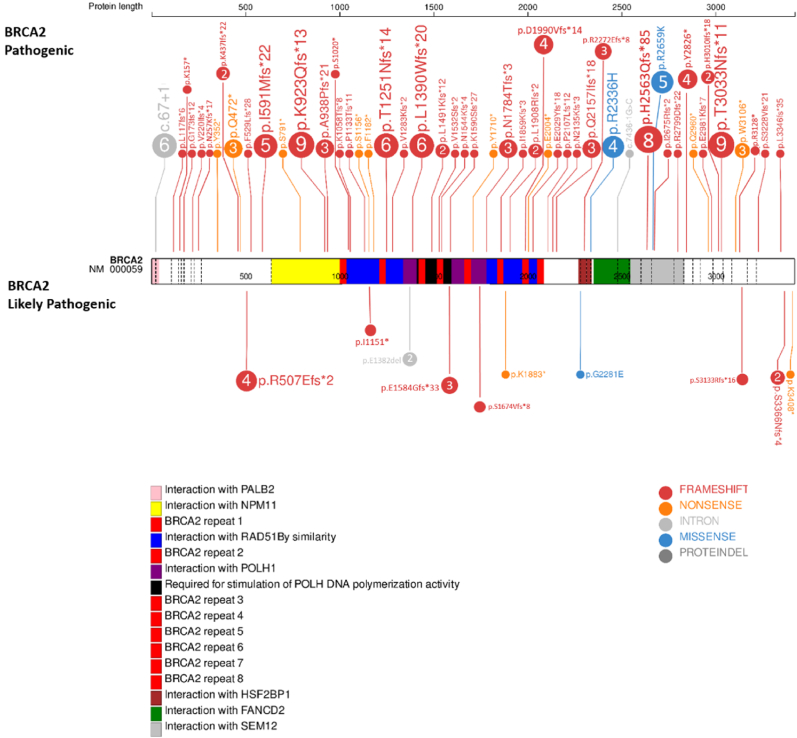
The detected pathogenic and likely pathogenic mutations in *BRCA2* gene (Created by using ProteinPaint, 2021) [44].

The most common variants detected in more than one patient in the *BRCA1* and *BRCA2* genes by region are shown in Table 5. Location and types of all detected clinically-relevant variants together with the affected domains in the *BRCA1* and *BRCA2* genes are shown in Fig. 3, Fig. 4.

**Table 5:** The most common *BRCA1*/2 variants detected by region.

Region	Gene	N	Genetic alteration		Variant Class.
Mediterranean Region	*BRCA1*	20	c.1444_1447delATTA	p.I482*	P
	*BRCA1*	6	c.5123C > A	p.A1708E	P
	*BRCA2*	9	c.3836A > G	p.N1279S	VUS
	*BRCA2*	6	c.4169delT	p.L1390Wfs*20	P
Marmara Region	*BRCA1*	7	c.5266dupC	p.Q1756Pfs*74	P
	*BRCA1*	4	c.181T > G	p.C61G	P
	*BRCA2*	3	c.67+1G > A	–	P
Aegean Region	*BRCA1*	7	c.5266dupC	p.Q1756Pfs*74	P
	*BRCA1*	4	c.181T > G	p.C61G	P
	*BRCA2*	3	c.67+1G > A	–	P
Central Anatolia Region	*BRCA1*	16	c.2800C > T	p.Q934*	P
	*BRCA1*	13	c.4327C > T	p.R1443*	P
	*BRCA1*	7	c.5266dupC	p.Q1756fs*74	P
	*BRCA2*	8	c.7689delC	p.H2563Qfs*85	P
	*BRCA2*	5	c.7976G > A	p.R2659K	P
Black Sea Region	*BRCA1*	3	c.6158 C > G	p.S2053C	VUS
Southeastern Anatolia Region	*BRCA1*	3	c.5266dupC	p.Q17756Pfs*74	P

## 4 Discussion

In this study, we performed *BRCA1*/2 genomic profiling on 2168 individuals from seven major regions across Turkey.

We observed significant differences even though the same enrollment and risk assessment criteria were followed. We also assume the imbalanced distribution of the number of patients is because of the imbalance in socio-economic status and the accessibility to health care services. We consider the high positivity rates in Aegean and Central Anatolian might be a result of regionally restricted but non-consanguineous marriages. Also eastern and southern regions had demographic differences due to the multiple population-based changes including immigration in recent years. On the other hand, Mediterranean, Marmara, Black Sea and Eastern Anatolia regions showed lower and similar positivity rates which might be a result of both geographical isolation and cultural differences.

The similar positivity rates between patients diagnosed with cancer and clinically unaffected individuals indicate that the risk assessments precised in terms of ACMG predisposition assessment criteria. So that the similar percentages could be due to patients' enrollment from high risk families.

Our results show discordance with the literature with respect to diagnosed patients' positivity rates (5–10%) and patients with hereditary cancers (20–25%) [15]. This indicates that most of our cancer patients likely carry inherited genetic alterations However, our screening positivity rates were lower than expected. We think that other cancer related genes may play an important role for cancer predisposition and progression, and should be considered for further investigation. Additionally, the positivity rates were similar with our previous study of 129 patients with a different cohort of breast cancer cases due to the only shared data focusing on selected Turkish cases independent of family history [16].

In contrast with the literature and our previous study, we found more clinically- relevant variations in the *BRCA2* gene than *BRCA1*. The majority of these *BRCA2* variants were classified as VUS and likely pathogenic rather than pathogenic [16]. We assume the reason is due to the lack of an in depth research on many of these Turkish-population-specific *BRCA* variants. However, we speculate that most of these VUS were detected in cancer patients may be supportive of their pathogenic potential. That's the reason of why all the most recent studies are focusing on the effects of rare variants that are hardly classified not only in cancer but also in rare diseases such as immunodeficiencies [[17], [18], [19]]. Even the VUSs have no functional studies supporting their pathogenicity, this study may provide as the first case series covering the all Turkey and a sourceful data for future functional studies or case based reportings and analysis.

Within the 471 detected variants there were 225 different genetic alterations of which 22 were novel. While 3 of these novel mutations were classified as pathogenic, 4 of them were likely pathogenic, 15 mutations were classified as VUS. The novel mutations detected in the study are shown in Table 6a, Table 6ba and 6b.

**Table 6a:** Novel pathogenic and likely pathogenic *BRCA1*/2 variants detected in the study.

Gene	Genetic alteration		Pathogenicity
*BRCA1*	c.2841delA	p.G948Efs*52	LP
	c.4070_4071delAA	p.E1357Gfs*10	P
	c.5057dupA	p.H1686Qfs*9	P
*BRCA2*	c.1519delA	p.R507Efs*2	LP
	c.4751del	p.E1584Gfs*33	LP
	c.5647A > T	p.K1883*	LP
	c.8020_8021dupAA	p.I2675Rfs*2	P

**Table 6b:** Novel VUS *BRCA1*/2 variants detected in the study.

Gene	Genetic alteration		Pathogenicity
*BRCA1*	c.5152 + 23C > T	–	VUS
*BRCA2*	c.1648G > A	p.E550K	VUS
	c.3239A > T	p.D1080V	VUS
	c.4766C > A	p.P1589Q	VUS
	c.5697T > A	p.D1899E	VUS
	c.6934G > C	p.D2312H	VUS
	c.6968A > C	p.H2323P	VUS
	c.7700A > G	p.Y2567C	VUS
	c.8021A > G	p.K2674R	VUS
	c.8332-47G > T	–	VUS
	c.8335T > G	p.S2779A	VUS
	c.8487 + 39T > C	–	VUS
	c.9370_9381delAACCTCCAGTGG	p.N3124_W3127del	VUS
	c.9370_9383delAACCTCCAGTGGCGinsCT	p.R3128delinsL	VUS
	c.9772G > A	p.E3258K	VUS

Moreover, none of the patients carrying more than one clinically significant mutation in either one of both *BRCA* genes had any Fanconi anemia history or findings when they were retrospectively examined and investigated in contrast with literature.

As the most striking outcome, our internal variant frequencies showed differences from the population databases that are in global use (including gnomAd, exAc, ESP, Allel Frequency Community and 1000 genomes). The majority of the most frequently reported variants in this study had significantly higher frequencies than in the global population. This shows the importance of establishing population-specific genomic databases for patient management and evaluation of an individual's susceptibility with regard to risk. Moreover, there were other studies reported the BRCA status of smaller and disease focused cohorts, our study is the first of its kind drawing the landscape all over the country showing the diversity in a regional aspect to pave the way for clinical interpretation of genetic testing and identifying the possible founder mutations.

The most frequent detected 3 variations of *BRCA1* and *BRCA2* genes compared with populational data in literature. The reported germline I482* variant in *BRCA1* gene was associated with familial ovarian cancer predisposition in German and Japanese populations [20,21]. Q1756fs*74 in *BRCA1* gene was also observed in Italian, Israel, USA, Brazilian, Ukrainian, Polish and Czech population with familial breast and ovarian cancer [[22], [23], [24], [25], [26], [27]]. The 3rd most frequent was Q934* which had been associated with epithelial ovarian cancer in Turkish population and; familial and non-familial ovarian cancer in Japanese and Argentinian [[28], [29], [30], [31]]. I482* variant was only detected in Mediterranean region; while Q934* and R1443* changes were observed only in Central Anatolia implicating that these variants can be speculated as founder mutations. However, further population studies must be carried out the support this conclusion.

One of the three most frequent variants in *BRCA2* gene, K923Qfs*13 mutation had only been reported in Evidence-based Network for the Interpretation of Germline Mutant Alleles (ENIGMA), while N1279S previously reported in Cypriot population with familial breast and/or ovarian cancer and in USA with breast cancer [[32], [33], [34]]. Finally, T3033Nfs*11 mutation was observed in Turkish, Caucasian and Ukrainian populations with familial breast and/or ovarian cancer; Denmark, China and Greek populations with breast cancer; Italian, Czech and Polish populations with hereditary high risk of breast/ovarian cancer [26,[35], [36], [37], [38], [39]].

In addition, C61G (observed in Marmara, Central Anatolia and Mediterranean regions) variant in BRCA1 gene was observed with significantly low allele frequency compared to global databases (0.1614%–0.6%) which also implicated in Saudi ovarian cancer patients. The variant had also been linked to hereditary breast and ovarian cancer in Italian and Polish populations [[40], [41], [42]]. On the contrary of global data (0.00199), p.A1708E variation detected in Mediterranean and Aegen regions in our study had higher allele frequency (0.1614) which is reported as the most frequent in Spanish and Latin populations.

The power of this article includes its large sample size that reflects a geographically diverse set of *BRCA1* and *BRCA2* mutation carriers. However, some limitations need to be considered. First, the sample set presented is not a systematic study of all Turkish populations or races/ethnicities; rather the data reflect patterns of recruitment of the consortium contributors' regions that they serve. Various sociodemographic groups and/or ethnic/racial groups that are over or under represented in our data set will, as a consequence, result in the mutational profiles reported be reflective of our convenience sample as opposed to the actual census. As a result, the most frequently observed mutations in some regions (e.g., Marmara Region) reflect the widespread use of this targeted testing panel rather than whole gene screening in the European population. Therefore, the relative frequencies of mutations by population in the present study may be subject to such testing biases. Comparing the relative frequencies is also complicated by the inclusion of related individuals. Secondly, although all the patients were enrolled via same criteria our analysis was based on self-reported geographical data of the study participants; this information however, may misclassify some groups of regions. For example, some Eastern Anatolia Region and Southeastern Anatolia Region groups may have been classified as Mediterranean or Marmara Region based on the data center available, but in fact may represent Eastern Anatolia Region and Southeastern Anatolia Region located citizens. This situation is due to a lack of well-equipped and experienced health centers in these regions. However, the regions' discrete variant size distribution is in harmony with latest general census. Finally, the frequency distributions of pathogenic and likely pathogenic variants of the world-wide population and our internal Turkish population are comparable. However, we see significant differences between the published VUS frequency distributions and our findings for the Turkish populations.

## 5 Conclusions

In conclusion, our internal variant frequencies showed differences from the population databases that are in global use. This indicates the significance of a population-specific genomic databases for patient management and evaluation of an individual's susceptibility.

## Author contributions

All authors have read and approved the final version of the manuscript, its content and its submission to the *The Breast*. AB, IB and OS drafted the original paper; AH, CM composed the figures; AB, SOS, MED, MSY, AAG, NA, OB, CDD, RE, SA, FSC, STB, SGT and MD edited and improved the content; AA, IB, BD, AH, CM, CO, SO, CR and OS performed data curation; AB, SGT and MD supervised the study; SOS, LA, MED, MSY, AAG, NA, OB, CDD, RE, SA, AA, OE, FSC, BD, STB, and MD commented on the manuscript.

## Declaration of competing of interest

The authors declare that they have no competing interests.
