# Supplementary material for: Germline landscape of BRCAs by 7-site collaborations as a BRCA consortium in Turkey
Source: Breast. 2022 Jun 21;65:15–22. doi: 10.1016/j.breast.2022.06.005 (PMC9249944; doi:10.1016/j.breast.2022.06.005)
Supplement: Multimedia component 1 [file mmc1.pdf]

Supplementary Table S1. Minor allele frequency comparison of detected variants in the *BRCA1* and *BRCA2* genes. Highest of global MAFs (gnomAd, exAc, ESP, Allel Frequency Community or 1000 genomes) were accepted for each alteration.

Gene	Genetic alteration	Pathogenicity Class.	n (allele)	Internal MAF %	Global MAF %
BRCA1	c.1444_1447delATTA p.I482*	P	20	0.4613	0
	c.5266dupC p.Q1756Pfs*74	P	17	0.3921	0.0183
	c.2800C>T p.Q934*	P	16	0.3690	0
	c.4327C>T p.R1443*	P	13	0.2998	0.00868
	c.181T>G p.C61G	P	7	0.1614	0.6
	c.5123C>A p.A1708E	P	7	0.1614	0.00199
	c.981_982delAT p.C328*	P	6	0.1384	0
	c.4035delA p.E1346fs*20	P	5	0.1153	0.00199
	c.2611_2612delCC p.P871Vfs*31	P	4	0.0923	0.00319
	c.3211G>T p.E1071*	P	4	0.0923	0
	c.3607C>T p.R1203*	P	4	0.0923	0.0012
	c.4391_4393delinsTT p.P1464Lfs*2	P	4	0.0923	0
	c.135-2A>T	P	3	0.0692	0
	c.3737C>A p.T1246N	VUS	3	0.0692	0
	c.1886G>T p.R629I	VUS	2	0.0461	0
	c.1895G>A p.S632N	VUS	2	0.0461	0
	c.2019delA p.E673Dfs*28	P	2	0.0461	0
	c.2666C>T p.S889F	VUS	2	0.0461	0.00119
	c.2952del p.I986Sfs*14	P	2	0.0461	0
	c.3328_3330delAAG p.K1110del	VUS	2	0.0461	0.0396
	c.3700_3704del p.V1234Qfs*8	P	2	0.0461	0.00318
	c.4063_4065delAAT p.N1355del	VUS	2	0.0461	0.000399
	c.4065_4068delTCAA p.N1355Kfs*10	P	2	0.0461	0.0012
	c.4070_4071delAA p.E1357Gfs*10	P	2	0.0461	0
	c.5057dupA p.H1686Qfs*9	P	2	0.0461	0
	c.509G>A p.R170Q	VUS	2	0.0461	0.00358
	c.5152+23C>T	VUS	2	0.0461	0
	c.535T>C p.Y179H	VUS	2	0.0461	0.000795
	c.53T>A p.M18K	LP	2	0.0461	0
	c.5444G>A p.W1815*	P	2	0.0461	0
	c.788dupG p.S264*	P	2	0.0461	0
	c.843_846delCTCA p.S282Yfs*15	P	2	0.0461	0
	c.2599C>G p.Q867E	VUS	2	0.0461	0
	c.5153-26A>G	VUS	2	0.0461	0
	c.692C>G p.T231R	VUS	1	0.0231	0
	c.1016delA p.K339Rfs*2	P	1	0.0231	0.00637
	c.1621C>T p.Q541*	P	1	0.0231	0
	c.1637_1685delinsGAAAG p.M546Ifs*5	P	1	0.0231	0
	c.1638_1685delinsAAAG p.M546Ifs*5	LP	1	0.0231	0
	c.1711A>G p.T571A	VUS	1	0.0231	0.000398
	c.1714G>T p.E572*	P	1	0.0231	0

	c.1938_1947delCAGTGAAGAG p.S646Rfs*2	P	1	0.0231	0
	c.1969C>T p.Q657*	P	1	0.0231	0
	c.2841delA p.G948Efs*52	LP	1	0.0231	0
	c.2959A>T p.K987*	P	1	0.0231	0
	c.2975delC p.T992fs*8	LP	1	0.0231	0
	c.3247A>G p.M1083V	VUS	1	0.0231	0
	c.3333delA p.E1112fs*5	P	1	0.0231	0
	c.3711A>G p.I1237M	VUS	1	0.0231	0.000398
	c.3756_3759delGTCT p.S1253fs*10	P	1	0.0231	0.00318
	c.3770_3771delAG p.E1257Gfs*9	P	1	0.0231	0.000796
	c.3825dupA p.L1276fs*11	LP	1	0.0231	0
	c.4185+21_4185+22dupTG	VUS	1	0.0231	0.00696
	c.4358-3A>G	VUS	1	0.0231	0
	c.4366A>G p.T1456A	VUS	1	0.0231	0
	c.4405C>A p.P1469T	VUS	1	0.0231	0
	c.4434G>C p.E1478D	VUS	1	0.0231	0
	c.4487C>A p.S1496*	P	1	0.0231	0
	c.4843G>A p.A1615T	VUS	1	0.0231	0.000398
	c.493_494delCT p.L165fs*16	P	1	0.0231	0
	c.4936del p.V1646Sfs*12	P	1	0.0231	0.000796
	c.4986+6T>G	LP	1	0.0231	0.0004
	c.4987A>T p.M1663L	VUS	1	0.0231	0
	c.5096G>A p.R1699Q	P	1	0.0231	0.00239
	c.5102_5103delTG p.L1722Qfs*14	P	1	0.0231	0.000398
	c.5194-2A>G	P	1	0.0231	0.000398
	c.536A>G p.Y179C	P	1	0.0231	0.0258
	c.923G>C p.S308T	VUS	1	0.0231	0.000796
	c.2765dupT p.K923Qfs*13	P	9	0.2076	0
	c.3836A>G p.N1279S	VUS	9	0.2076	0
	c.9097dupA p.T3033Nfs*11	P	9	0.2076	0.00122
	c.7689delC p.H2563Qfs*85	P	8	0.1845	0
	c.8881G>A p.G2961S	VUS	8	0.1845	0
	c.3318C>G p.S1106R	VUS	7	0.1614	0.00042
	c.3751dupA p.T1251Nfs*14	P	6	0.1384	0.000407
	c.4169delT p.L1390Wfs*20	P	6	0.1384	0
	c.67+1G>A	P	6	0.1384	0
	c.7976G>A p.R2659K	P	5	0.1153	0.000398
	c.1773_1776delTTAT p.I591Mfs*22	P	5	0.1153	0.00121
	c.1411G>A p.E471K	VUS	4	0.0923	0
	c.5969delA p.D1990Vfs*14	P	4	0.0923	0
	c.7007G>A p.R2336H	P	4	0.0923	0
	c.7472A>T p.Q2491L	VUS	4	0.0923	0.000795
	c.8478C>A p.Y2826*	P	4	0.0923	0.000398
	c.1519delA p.R507Efs*2	LP	4	0.0923	0
	c.1414C>T p.Q472*	P	3	0.0692	0
	c.2808_2811delACAA p.A938Pfs*21	P	3	0.0692	0.000797
	c.4751del p.E1584Gfs*33	LP	3	0.0692	0
**BRCA2**	c.5351dupA p.N1784Tfs*3	P	3	0.0692	0

	c.6158C>G p.S2053C	VUS	3	0.0692	0
	c.6468_6469delTC p.Q2157Ifs*18	P	3	0.0692	0.000436
	c.6814delA p.R2272Efs*8	P	3	0.0692	0
	c.9052_9057delAGTAAA p.K3019_3020del	VUS	3	0.0692	0.00279
	c.9317G>A p.W3106*	P	3	0.0692	0
	c.10095delCinsGAATTATATCT p.S3366Nfs*4	LP	2	0.0461	0.0637
	c.1235C>G p.P412R	VUS	2	0.0461	0
	c.1310_1313delAAGA p.K437Ifs*22	P	2	0.0461	0.000411
	c.1343G>A p.R448H	VUS	2	0.0461	0.000403
	c.1909+22delT	VUS	2	0.0461	11.3
	c.2264C>G p.S755C	VUS	2	0.0461	0
	c.3073A>G p.K1025E	VUS	2	0.0461	0.0048
	c.3503T>C p.M1168T	VUS	2	0.0461	0.000399
	c.4081C>G p.Q1361E	VUS	2	0.0461	0
	c.4146_4148delAGA p.E1382del	LP	2	0.0461	0.00733
	c.4446_4451dupAACAGA p.E1482_T1483dup	VUS	2	0.0461	0.0004
	c.5722_5723delCT p.L1908Rfs*2	P	2	0.0461	0.000399
	c.6080G>A p.R2027K	VUS	2	0.0461	0
	c.6550C>G p.Q2184E	VUS	2	0.0461	0.00122
	c.6935A>T p.D2312V	VUS	2	0.0461	0.0244
	c.7435+10G>A	VUS	2	0.0461	0.0004
	c.7645T>G p.C2549G	VUS	2	0.0461	0
	c.9027delT p.H3010Ifs*18	P	2	0.0461	0
	c.9501+4A>G	VUS	2	0.0461	0.00119
	c.9586A>G p.K3196E	VUS	2	0.0461	0.00994
	c.5836T>C p.S1946P	VUS	2	0.0461	0.00159
	c.6008T>C p.I2003T	VUS	2	0.0461	0
	c.9649-37A>C	VUS	2	0.0461	0.000431
	c.9839C>A p.P3280H	VUS	2	0.0461	0.00159
	c.10037_10046delTGATAAATACinsATT p.L3346fs*35	P	1	0.0231	0.00318
	c.10078A>G p.K3360E	VUS	1	0.0231	0
	c.10222A>T p.K3408*	LP	1	0.0231	0.00318
	c.1055dupA p.Y352*	P	1	0.0231	0
	c.1146A>T p.K382N	VUS	1	0.0231	0.000399
	c.155A>G p.H52R	VUS	1	0.0231	0.000398
	c.1587_1590delTAAA p.F529Lfs*28	P	1	0.0231	0
	c.1592A>C p.K531T	VUS	1	0.0231	0
	c.1627C>A p.H543N	VUS	1	0.0231	0
	c.1648G>A p.E550K	VUS	1	0.0231	0
	c.1951G>T p.D651Y	VUS	1	0.0231	0.000416
	c.2372C>A p.S791*	P	1	0.0231	0
	c.2731G>C p.E911Q	VUS	1	0.0231	0
	c.280C>T p.P94S	VUS	1	0.0231	0.00478
	c.2892A>T p.K964N	VUS	1	0.0231	0.00443
	c.2918C>G p.S973W	VUS	1	0.0231	0.000402

c.3031A>G p.T1011A	VUS	1	0.0231	0
c.3059_3060delCT p.S1020*	P	1	0.0231	0
c.3171_3172del p.K1058Tfs*8	P	1	0.0231	0
c.3239A>T p.D1080V	VUS	1	0.0231	0
c.3302A>G p.H1101R	VUS	1	0.0231	0
c.3396dupA p.P1133Tfs*11	P	1	0.0231	0
c.3449_3452delCTAT p.I1151*	LP	1	0.0231	0
c.3465_3466delTT p.S1156*	P	1	0.0231	0.000398
c.349_350delCT p.L117fs*6	P	1	0.0231	0
c.3545_3546delTT p.F1182*	P	1	0.0231	0
c.3599G>C p.C1200S	VUS	1	0.0231	0
c.375T>A p.D125E	VUS	1	0.0231	0
c.3847_3848delGT p.V1283Kfs*2	P	1	0.0231	0.00538
c.3854_3856delAAA p.K1286del	VUS	1	0.0231	0.00869
c.385G>T p.D129Y	VUS	1	0.0231	0
c.3894T>G p.I1298M	VUS	1	0.0231	0
c.4237A>G p.K1413E	VUS	1	0.0231	0
c.4243G>C p.E1415Q	VUS	1	0.0231	0.000412
c.4470_4473delACTG p.L1491Kfs*12	P	1	0.0231	0.000399
c.4471_4474delCTGA p.L1491Kfs*12	P	1	0.0231	0.000399
c.4531G>A p.E1511K	VUS	1	0.0231	0.0028
c.4587_4588insA p.V1532Sfs*2	P	1	0.0231	0.0004
c.4631dupA p.N1544Kfs*4	P	1	0.0231	0.0004
c.469A>T p.K157*	P	1	0.0231	0
c.4766C>A p.P1589Q	VUS	1	0.0231	0
c.4769delA p.K1590Sfs*27	P	1	0.0231	0.000399
c.4928T>C p.V1643A	VUS	1	0.0231	0.00287
c.5020delA p.S1674Vfs*8	LP	1	0.0231	0.000399
c.5130_5133delTGTA p.Y1710*	P	1	0.0231	0.7
c.518delG p.G173fs*12	P	1	0.0231	0.00319
c.5331_5333delGAA p.K1777del	VUS	1	0.0231	0
c.5483A>G p.K1828R	VUS	1	0.0231	0
c.5576_5579del p.I1859Kfs*3	P	1	0.0231	0
c.5590G>A p.D1864N	VUS	1	0.0231	0.00122
c.5647A>T p.K1883*	LP	1	0.0231	0
c.5697T>A p.D1899E	VUS	1	0.0231	0
c.575T>C p.M192T	VUS	1	0.0231	0
c.5860A>G p.T1954A	VUS	1	0.0231	0
c.5870T>C p.I1957T	VUS	1	0.0231	0.00239
c.5975C>T p.S1992L	VUS	1	0.0231	0.000399
c.6010G>T p.E2004*	P	1	0.0231	0.000399
c.6085_6089delGAAAA p.E2029Yfs*18	P	1	0.0231	0.000797
c.6106C>T p.P2036S	VUS	1	0.0231	0.000797
c.6231G>C p.K2077N	VUS	1	0.0231	0.0117
c.6320delC p.P2107Lfs*12	P	1	0.0231	0
c.6365T>C p.M2122T	VUS	1	0.0231	0
c.6405_6409delCTTAA p.N2135Kfs*3	P	1	0.0231	0.000416
c.6469C>T p.Q2157*	P	1	0.0231	0
c.658_659delGT p.V220Ifs*4	P	1	0.0231	0.00449

	c.6614T>G p.V2205G	VUS	1	0.0231	0
	c.6742C>A p.H2248N	VUS	1	0.0231	0.000398
	c.6842G>A p.G2281E	LP	1	0.0231	0.000408
	c.6934G>C p.D2312H	VUS	1	0.0231	0
	c.6968A>C p.H2323P	VUS	1	0.0231	0
	c.7072T>C p.S2358P	VUS	1	0.0231	0.000797
	c.7436-1G>C	P	1	0.0231	0
	c.7522G>A p.G2508S	VUS	1	0.0231	0.0159
	c.7633G>A p.V2545I	VUS	1	0.0231	0.000399
	c.7700A>G p.Y2567C	VUS	1	0.0231	0
	c.771_775delTCAAA p.N257Kfs*17	P	1	0.0231	0.000798
	c.7723A>C p.T2575P	VUS	1	0.0231	0
	c.7766C>T p.P2589L	VUS	1	0.0231	0.000795
	c.7783G>T p.A2595S	VUS	1	0.0231	0
	c.7855T>C p.W2619R	VUS	1	0.0231	0
	c.7883T>C p.I2628T	VUS	1	0.0231	0
	c.8020_8021dupAA p.I2675Rfs*2	P	1	0.0231	0
	c.8021A>G p.K2674R	VUS	1	0.0231	0
	c.8092G>A p.A2698T	VUS	1	0.0231	0.00358
	c.8117A>G p.N2706S	VUS	1	0.0231	0.00716
	c.8155A>G p.I2719V	VUS	1	0.0231	0
	c.8332-47G>T	VUS	1	0.0231	0
	c.8324T>G p.M2775R	VUS	1	0.0231	0.00318
	c.8335T>G p.S2779A	VUS	1	0.0231	0
	c.8359C>T p.R2787C	VUS	1	0.0231	0.000398
	c.8395delA p.R2799Dfs*22	P	1	0.0231	0
	c.8452G>A p.V2818I	VUS	1	0.0231	0.000398
	c.8487+39T>C	VUS	1	0.0231	0
	c.8878C>T p.Q2960*	P	1	0.0231	0
	c.8897T>C p.V2966A	VUS	1	0.0231	0.0004
	c.8940delA p.E2981Kfs*7	P	1	0.0231	0
	c.8953+80G>A	VUS	1	0.0231	0.0127
	c.9370_9381delAACCTCCAGTGG<br>p.N3124_W3127del	VUS	1	0.0231	0
	c.9370_9383delAACCTCCAGTGGCGinsCT<br>p.R3128delinsL	VUS	1	0.0231	0
	c.9373C>T p.L3125F	VUS	1	0.0231	0
	c.9382C>T p.R3128*	P	1	0.0231	0.00119
	c.9397_9398delTC p.S3133Rfs*16	LP	1	0.0231	0
	c.9613_9614delGCinsCT p.A3205L	VUS	1	0.0231	0.00239
	c.9682delA p.S3228Vfs*21	P	1	0.0231	0.0004
	c.9730G>T p.V3244F	VUS	1	0.0231	0.00119
	c.9772G>A p.E3258K	VUS	1	0.0231	0
	c.995T>A p.I332N	VUS	1	0.0231	0
