# Supplementary material for: Germline landscape of BRCAs by 7-site collaborations as a BRCA consortium in Turkey
Source: Breast. 2022 Jun 21;65:15–22. doi: 10.1016/j.breast.2022.06.005 (PMC9249944; doi:10.1016/j.breast.2022.06.005)
Supplement: Multimedia component 2 [file mmc2.docx]

| Diagnosis | Mediterranean  Region | Aegean  Region | Black Sea  Region | Central Anatolia  Region | Marmara  Region | Eastern Anatolia  Region | Southeastern Anatolia  Region | Total |
| --- | --- | --- | --- | --- | --- | --- | --- | --- |
| Breast cancer | 608 | 46 | 65 | 111 | 323 | 49 | 21 | 1223 |
| Breast and endometrium cancer | 3 | - | 1 | - | 4 | - | 1 | 9 |
| Breast and ovarian cancer | 172 | - | 1 | 4 | 10 | - | - | 187 |
| Cervical cancer | 3 | - | 1 | - | 3 | - | - | 7 |
| Endometrium cancer | 21 | - | 1 | - | 5 | - | - | 27 |
| Ovarian cancer | 76 | 5 | 10 | 34 | 59 | 9 | 3 | 196 |
| Over and endometrium cancer | 4 | - | 1 | - | 1 | - | - | 6 |
| Total | *897* | *51* | *80* | *149* | *405* | *58* | *25* | *1655* |
| Screening | 369 | 2 | 9 | 79 | 49 | 4 | 1 | 513 |
| Overall total | *1256* | *53* | *89* | *228* | *454* | *62* | *26* | *2168* |

**Supplementary Table S2.** The number of affected and unaffected individuals and region-based clinical distribution of affected patients.
